# DNA methylation dynamic of bone marrow hematopoietic stem cells after allogeneic transplantation

**DOI:** 10.1186/s13287-019-1245-6

**Published:** 2019-05-20

**Authors:** Stefania Trino, Pietro Zoppoli, Angelo Michele Carella, Ilaria Laurenzana, Alessandro Weisz, Domenico Memoli, Giovanni Calice, Francesco La Rocca, Vittorio Simeon, Lucia Savino, Luigi Del Vecchio, Pellegrino Musto, Antonella Caivano, Luciana De Luca

**Affiliations:** 1 Laboratory of Preclinical and Translational Research, IRCCS - Referral Cancer Center of Basilicata (CROB), 85028 Rionero in Vulture, Italy; 2 SSD Unità di terapia intensiva ematologica e terapie cellulari, Fondazione IRCCS-Casa Sollievo della Sofferenza, San Giovanni Rotondo, Italy; 3 Laboratory of Molecular Medicine and Genomics, Department of Medicine, Surgery and Dentistry Scuola Medica Salernitana, University of Salerno, Baronissi, SA Italy; 4 Laboratory of Clinical Research and Advanced Diagnostics, IRCCS - Referral Cancer Center of Basilicata (CROB), 85028 Rionero in Vulture, Italy; 5 Medical Statistics Unit, University of Campania “Luigi Vanvitelli”, Naples, Italy; 6 Department of Molecular Medicine and Medical Biotechnologies, University of Naples Federico II, 80138 Naples, Italy; 7 Unit of Hematology and Stem Cell Transplantation, IRCCS - Referral Cancer Center of Basilicata (CROB), 85028 Rionero in Vulture, Italy

**Keywords:** Allogeneic hematopoietic bone marrow stem cell transplantation, Hematopoietic stem and progenitor cells, DNA methylation, CpG sites, Hematological malignancies, Promoter methylation region

## Abstract

**Background:**

Allogeneic hematopoietic stem cell transplantation (AHSCT) is a curative therapeutic approach for different hematological malignancies (HMs), and epigenetic modifications, including DNA methylation, play a role in the reconstitution of the hematopoietic system after AHSCT. This study aimed to explore global DNA methylation dynamic of bone marrow (BM) hematopoietic stem and progenitor cells (HSPCs) from donors and their respective recipients affected by acute myeloid leukemia (AML), acute lymphoid leukemia (ALL) and Hodgkin lymphoma (HL) during the first year after transplant.

**Methods:**

We measured DNA methylation profile by Illumina HumanMethylationEPIC in BM HSPC of 10 donors (t0) and their matched recipients at different time points after AHSCT, at day + 30 (t1), + 60 (t2), + 120 (t3), + 180 (t4), and + 365 (t5). Differential methylation analysis was performed by using R software and CRAN/Bioconductor packages. Gene set enrichment analysis was carried out on promoter area of significantly differentially methylated genes by clusterProfiler package and the mSigDB genes sets.

**Results:**

Results show significant differences in the global methylation profile between HL and acute leukemias, and between patients with mixed and complete chimerism, with a strong methylation change, with prevailing hyper-methylation, occurring 30 days after AHSCT. Functional analysis of promoter methylation changes identified genes involved in hematopoietic cell activation, differentiation, shaping, and movement. This could be a consequence of donor cell “adaptation” in recipient BM niche. Interestingly, this epigenetic remodeling was reversible, since methylation returns similar to that of donor HSPCs after 1 year. Only for a pool of genes, mainly involved in dynamic shaping and trafficking, the DNA methylation changes acquired after 30 days were maintained for up to 1 year post-transplant. Finally, preliminary data suggest that the methylation profile could be used as predictor of relapse in ALL.

**Conclusions:**

Overall, these data provide insights into the DNA methylation changes of HSPCs after transplantation and a new framework to investigate epigenetics of AHSCT and its outcomes.

**Electronic supplementary material:**

The online version of this article (10.1186/s13287-019-1245-6) contains supplementary material, which is available to authorized users.

## Background

Epigenetic regulation, including DNA methylation, histone modification, chromatin remodeling, and noncoding RNA regulation, has been reported to regulate gene expression [1, 2]. One important hallmark of the epigenome is its great plasticity in response to internal (i.e., during development and transplant) and environmental factors [3]. In fact, this process is important for normal biological functions like immune cell development and differentiation [4] and for tumor conditions [3].

DNA methylation is a reversible process of attaching methyl residues to cytosines adjacent to guanines (CpGs) [5]. CpGs are distributed throughout the whole genome, including repetitive sequences, enhancers, promoters, and gene body [6]. DNA methylation had deep effects on gene expression by influencing the accessibility of transcription factors to DNA, altering genetic stability and modifying genomic structure [7, 8]. Specifically, the methylation of promoter CpGs is associated with a stable gene silencing, and its dysregulation plays an important role in oncogenesis and tumor progression [9]. On the other hand, the methylation of CpGs in gene body increased gene expression [10]. CpGs are densely clustered in regions called CpG islands in which impact on gene expression is still unclear. Overall, it seems to contribute significantly to global gene expression regulation specifically if CpG islands are located in promoter regions [6, 11, 12]. Nowadays, regions with relatively lower CpG density are gaining importance in DNA methylation studies. In fact, methylation status of the majority of CpG islands across a variety of tissues and cell populations is non-dynamic and less variant [13–15]. It is now proven, on the contrary, that methylation is dynamic along the CpG shores (< 2 kb flanking CpG Islands), CpG shelves (< 2 kb flanking outwards from a CpG shore), and open sea (outside of the CpG island/shores/shelves context). Recent works, in fact, have shown that DNA methylation of shore and shelf in intergenic region was associated with increased gene expression [14, 16] and a hypo-methylation of open sea with a transcriptional silencing [17].

In normal hematopoiesis and in hematological malignancies (HMs), epigenetic modifications [18, 19], including DNA methylation, play an important role in self-renewal of stem cells, in differentiation and in the malignancy pathogenesis [20].

In HMs, bone marrow (BM) transplantation, is an important treatment choice, which allows the restoration of blood cellular components [21]. In particular, the allogeneic hematopoietic stem cell transplantation (AHSCT) is a curative therapeutic approach for leukemia, lymphoma, multiple myeloma, and myeloproliferative disease [4, 22]. This therapy consists of the intravenous infusion of hematopoietic stem and progenitor cells (HSPCs) to reestablish marrow function in patients with damaged or defective BM [23]. The sources of HSPCs include human leukocyte antigen (HLA)-matched siblings, matched unrelated donors, unrelated umbilical cord blood (UCB), and HLA haplotype-mismatched donors (HLA-haploidentical) [24, 25]. After conditioning regimen (myeloablative, reduced intensity conditioning, or non-myeloablative) [26, 27], HM patients received HSPCs from mobilized peripheral blood (PB), BM, or UCB of donors.

The process through which transplanted stem cells reach the BM and begin to produce healthy blood cells is called engraftment phase and approximately occurs from 2 to 4 weeks [28, 29]. The first sign of engraftment is the gradual rise of both white blood cell and platelet count that begins about 3 weeks after transplant. Red blood cells often take a little longer to begin developing [26]. Another routine diagnostic tool for the assessment of engraftment and early detection of graft failure is the analysis of chimerism in PB cells [30]. A full chimerism is achieved when more than 95% of cells derives from the donor. Instead, mixed chimerism is defined as having 5–95% recipient-derived hematopoietic cells remaining [31]. The epigenetic modifications, such as DNA methylation, play a critical role in self-renewal and in differentiation of HSPCs [4], but little is known about their changes on hematopoietic cells during transplant. In this context, this study analyzed, for the first time, the DNA methylation dynamic of HSPCs from donors and from HM patients during the time of AHSCT, from 30 days to 1 year, using a genome-wide approach.

## Methods

### Study samples

From March 2013 to March 2015, a total of 10 donors and their respective 10 patients who received BM AHSCT were included in our study and followed up to a maximum of 55 months after transplant. BM samples were provided by the Department of Hematology and Stem Cell Transplantation Unit, IRCCS “Casa Sollievo della Sofferenza” Hospital, San Giovanni Rotondo, Italy. BM samples were sequentially collected from donors (t0) and matched recipients at different time points, at day + 30 (t1), + 60 (t2), + 120 (t3), + 180 (t4), and + 365 (t5). All participants gave written informed consent in accordance with the Declaration of Helsinki. Patient and donor characteristics are shown in Table 1. Peripheral blood was evaluated after transplantation to identify the presence of mixed or full chimerism by the analysis of genomic polymorphisms.

**Table 1:** Characteristics of patients and donors

Characteristics	
Total patients, *n*	10
Sex, male, *n* (%)	3 (30%)
Age, mean (range) yr.	34 (17–57)
Diagnosis	
AML, *n* (%)	6 (60%)
ALL, *n* (%)	3 (30%)
HL, *n* (%)	1 (10%)
Conditioning regimen	
Myeloablative, *n* (%)	9 (90%)
Reduced Intensity Conditioning, *n* (%)	1 (10%)
Chimerism	
Complete, *n* (%)	9 (90%)
Mixed, *n* (%)	1 (10%)
4-years survival, *n* (%)	8 (80%)
Donor sex, male, *n* (%)	6 (60%)
Donor sex, female, *n* (%)	4 (40%)
Donor age, mean (range)	33 (14–52)
Stem cell source (%)	
BM, *n* (%)	10 (100%)
Donor type	
Matched sibling, *n* (%)	4 (40%)
Matched unrelated, *n* (%)	3 (30%)
Mismatched haploidentical, *n* (%)	3 (30%)

### Human CD34^+^ HSPCs isolation

BM mononuclear cells from donors and patients were obtained by Ficoll-Paque gradient centrifugation. CD34^+^ cells were isolated from mononuclear cells by CD34 Microbead Kit (Miltenyi Biotec, Auburn, CA). The purity of isolated CD34^+^ cells, verified by flow cytometry, routinely ranged between 90 and 95%.

### Genomic DNA isolation

DNA was extracted from CD34^+^ cell population by AllPrep DNA/RNA Micro Kit (Qiagen GmbH, Hilden, Germany). DNA quality was controlled by agarose gel electrophoresis and quantified by a NanoDrop ND-1000 Spectrometer (Thermo Scientific, Wilmington, DE, USA).

### Bisulfite conversion and array-based DNA methylation

Genomic DNA (250 ng) was treated with sodium bisulfite using the Zymo EZ DNA Methylation Kit (Zymo Research, Orange, CA, USA) according to the manufacturer’s procedure, with the alternative incubation conditions recommended when using the Illumina Infinium Methylation Assay. The methylation assay was performed on 4 μl bisulfite-converted genomic DNA at 50 ng/μl according to the Infinium HD Methylation Assay protocol (Illumina, CA, USA). The bisulfite-converted genomic DNA was amplified at 37 °C for 22 h, enzymatically fragmented, purified, and hybridized on an Infinium HumanMethlyationEPIC (850k) BeadChip at 48 °C for 17 h. The BeadChip was then washed to remove any un-hybridized or non-specific hybridized DNA. Labeled single-base extension was performed on primers hybridized with DNA, and the hybridized DNA was removed. The extended primers were stained with multiple layers of fluorescence; the BeadChip was then coated using a proprietary solution and scanned using the Illumina HiScanSQ system (Illumina).

### Microarray data analysis

Methylation signals were analyzed as described in Pistore et al. [32], and all other statistical analyses were performed using R software [33] and CRAN/Bioconductor packages.

The methylation level for each cytosine was expressed as beta value (the ratio of the fluorescence intensity of the methylated to unmethylated versions of the probes) as well as *M* values (log2 ratio of the intensities of methylated probe versus unmethylated probe). Although the beta value has a more intuitive biological interpretation, the *M* value is considered more valid statistically [34], so we used this for statistical analysis and the beta value for data description and plotting.

Overlap analysis of methylation EPIC probes with genomic features (such as TSS1500, TSS200, 1st Exon, 5′UTR, Gene body, 3′UTR, and IGR) and with CpG localization (islands, Shores, Shelves, or Open Sea) was determined exploiting the annotation stored in the Illumina’s EPIC methylation arrays Bioconductor package [35].

Multdimensional scaling (or principle coordinate analysis) and clustering analysis performed on the methylation level (*M* value) of the most variable probes. For each sample, we also evaluated the “promoter” and “body” regions’ mean methylation level. The methylation profile of CD34 gene was further evaluated.

Differential methylation analysis was performed using limma, minfi, and DMRcate packages [36–38]. Differentially methylated probes (DMPs) and differentially methylated regions (DMRs) specifically annotated for gene region and CGI position and their relative distribution (as probes, genes, and regions) were analyzed. Probes and regions with absolute FC (*M* value) greater than 1.5 (abs (log2(*M* value) > 0.58)) with adjusted (fdr) *p* value < 0.05 were considered significant.

To better understand stable methylation modifications in the promoter area, we defined genes significantly and concordant differentially methylated in t1 versus t0 and t5 versus t0 as “stable,” while the discordant genes are labeled as “revert.” We also searched for these “stable” genes in t2 vs t0. Significant gene sets (*p* value < 0.05) were obtained by gene set enrichment analysis (clusterProfiler package [39] and the mSigDB genes sets [40]).

## Results

### Analysis of global DNA methylation profile in both donor and recipient HSPCs

**Fig. 1:**
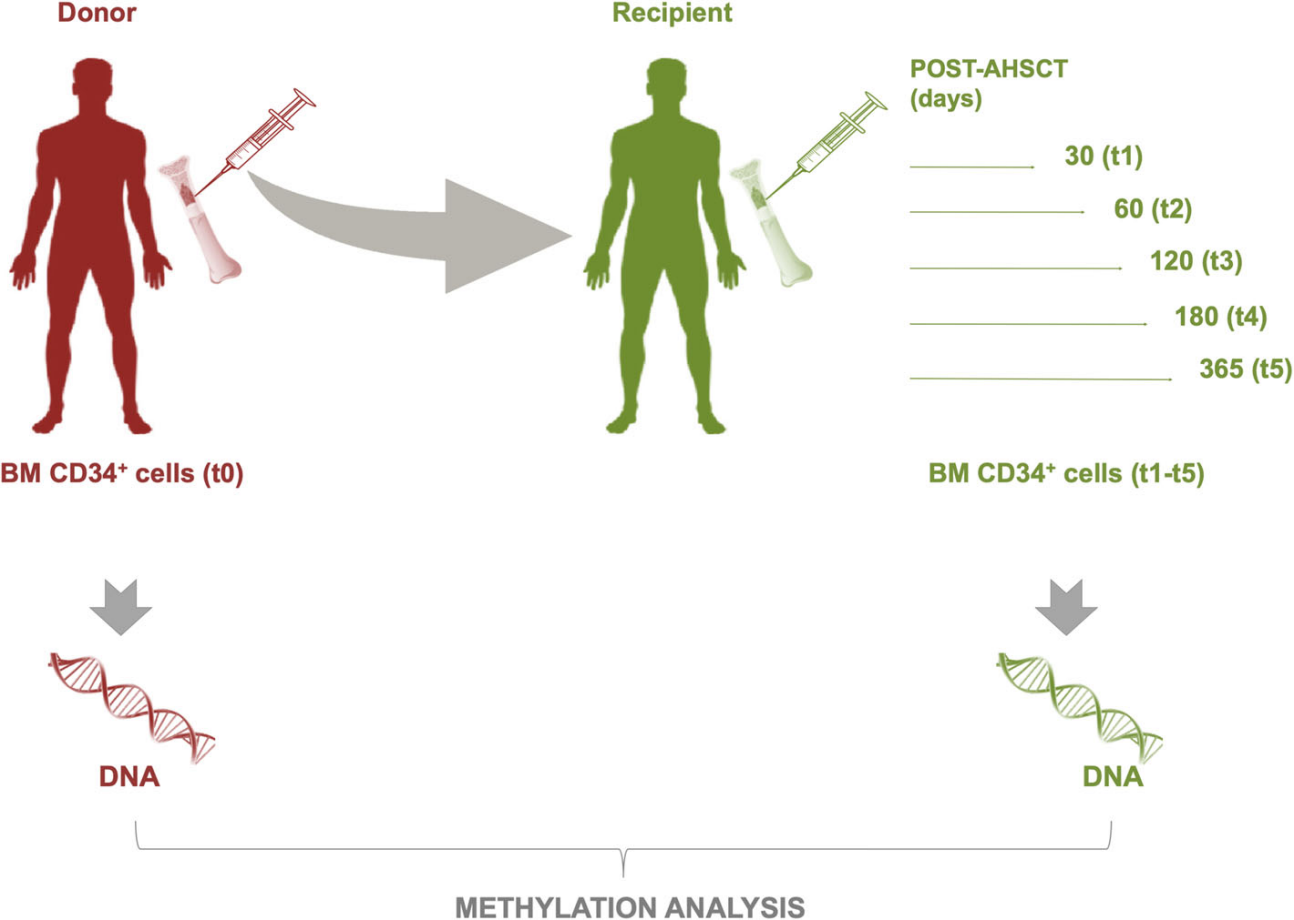
Diagram of experimental design. Analysis of genome-wide DNA methylation was performed on BM CD34^+^ cells of donors (t0) and recipients at different points after transplant + 30 (t1), + 60 (t2), + 120 (t3), + 180 (t4), and + 365 (t5)

Global methylation profiles were investigated in BM HPSCs purified as CD34^+^ cells from 10 donors (t0) and 10 respective HM recipients [3 acute lymphoid leukemia (ALL), 6 acute myeloid leukemia (AML), and 1 Hodgkin lymphoma (HL)] in sequential time points [30 (t1), 60 (t2), 120 (t3), 180 (t4), and 365 (t5) days] after AHSCT. A schematic overview of the study design was reported in Fig. 1. We used the Illumina Infinium MethylationEPIC (850k) arrays containing over 850,000 probes which cover the broad content categories including the following: CpG island, North (N) and South (S) shores and shelves, open sea, non CpG methylated sites, FANTOM enhancers, ENCODE open chromatin and enhancers, DNA hypersensitivity sites, and miRNA promoter regions. EPIC probes are located at transcription start site (TSS) 1500, TSS 200, 5′ untranslated region (UTR), first exon, gene body, 3′UTR, exon boundaries, and intergenic regions.

**Fig. 2:**
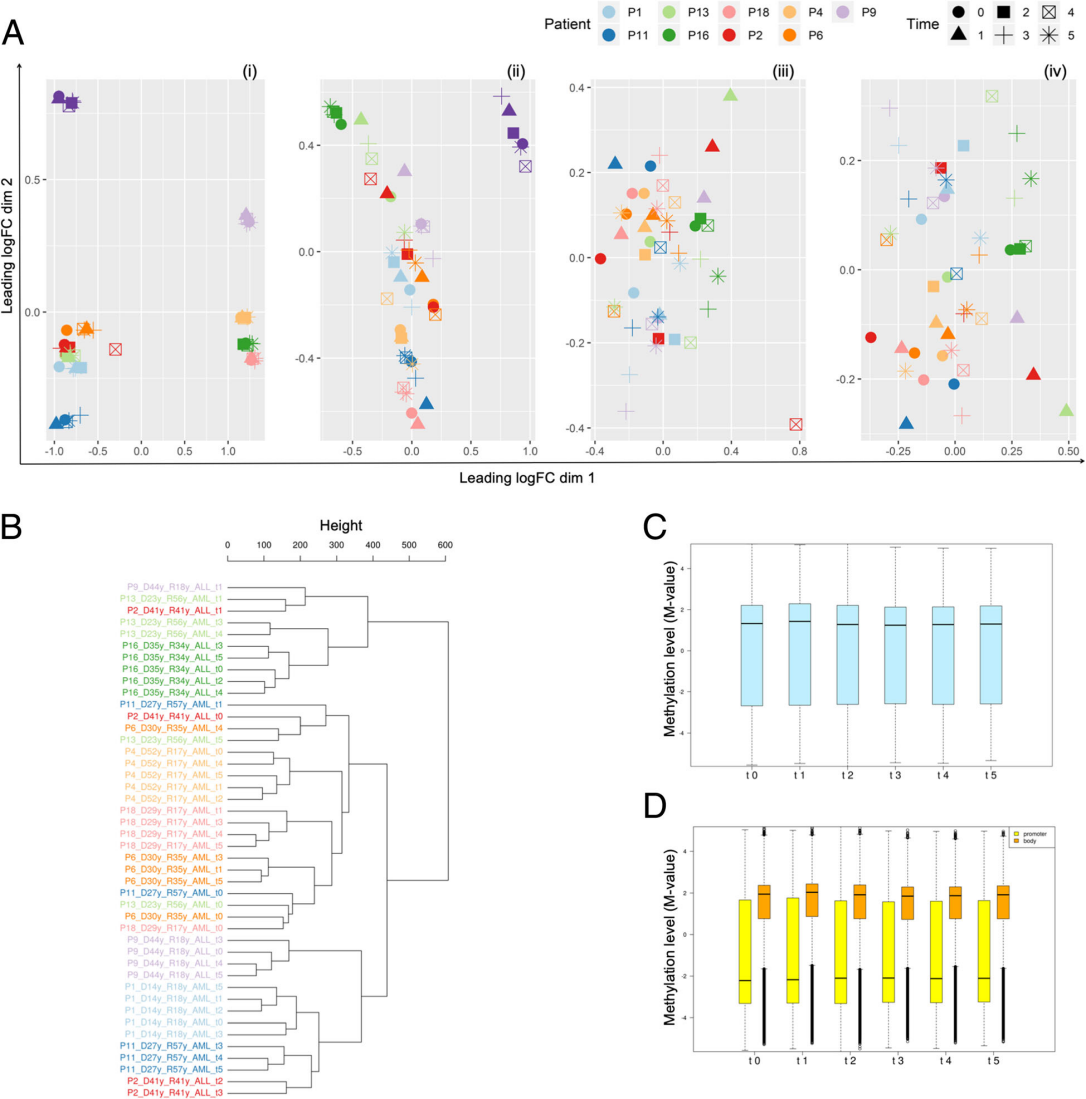
Analysis of global DNA methylation profile. **a** Multidimensional scaling plot of donor (P0) and patient samples (Pn); assessment and elimination of confounding factors: (i) segregation of patients due to sex (elimination of probes on sex chromosomes), (ii) segregation of P19 cluster (t0-t5) from all other samples (elimination of P19 samples), (iii) segregation of P2 t4 from all other samples (elimination of P2 t4), (iv) no other confounding factors found. **b** Unsupervised hierarchical clustering of global methylation profile on the most variable probes; for each sample, the figure indicated patient number (P), donor age (D years) and recipient age (R years), disease (AML or ALL), and respective time points (t0 or t1-t5). Each patient with its donor was annotated with a specific color. **c** Global DNA methylation profile (*M* value) in grouped donors (t0) and patients in all time points (t1, t2, t3, t4, t5). **d** Global DNA methylation profile (*M* value) of promoter (yellow) and gene body (orange) regions in grouped donors (t0) and patients in all time points (t1, t2, t3, t4, t5). Horizontal bar indicated the median of methylation

In order to identify potential confounding factors in methylation data, we performed a multidimensional scaling (MDS) on the probes with the largest standard deviations between samples [36]. As showed in Fig. 2a, MDS segregated in distinct clusters along the principal component 1: (i) male and female, (ii) all time points after AHSCT of patient 19 (t1-t5 P19) with its donor (t0 P19) and the other HM patients with respective donors, and finally (iii) t4 of patient 2 (P2_4) and all time points of HM patients with respective donors. Eliminating those confounding factors (sex chromosomes, t0 and t1-t5 P19, and P2_4), a uniform distribution of DNA methylation was observed among all samples (Fig. 2a, iv).

Unsupervised hierarchical clustering of global methylation profile on the most variably probes showed that t1-t5 of most patients (P1, P4, P6, P9, P11, P16, and P18) profiled into a specific methylation cluster. Moreover, analyzing the donor (t0) distribution, we observed that four donors (P11_t0, P13_t0, P6_t0, and P18_t0) clustered together, other four (P1_t0, P4_t0, P9_t0, and P16_t0) clustered with t1-t5 of respective recipients, and just one (P2_t0) segregated separately (Fig. 2b). To evaluate the potential global methylation change in donors and patients after AHSCT, we examined global level (*M* value) in both grouped donors (t0) and HM patients in all time points (t1-t5) discovering a similar median of methylation in all groups with a global hyper-methylation dominance (Fig. 2c). Specifically, distinguishing the genome in promoter (TSS200, TSS1500, 5′UTR) and body (1st exon, gene bodies, 3′UTR, exon boundaries) regions, we observed that global methylation levels of both regions were similar from t0 to t5 with a low methylation level in promoter region and, conversely, a hyper-methylation in gene body (Fig. 2d).

### Differential methylation analysis between donor and recipient HSPCs

**Fig. 3:**
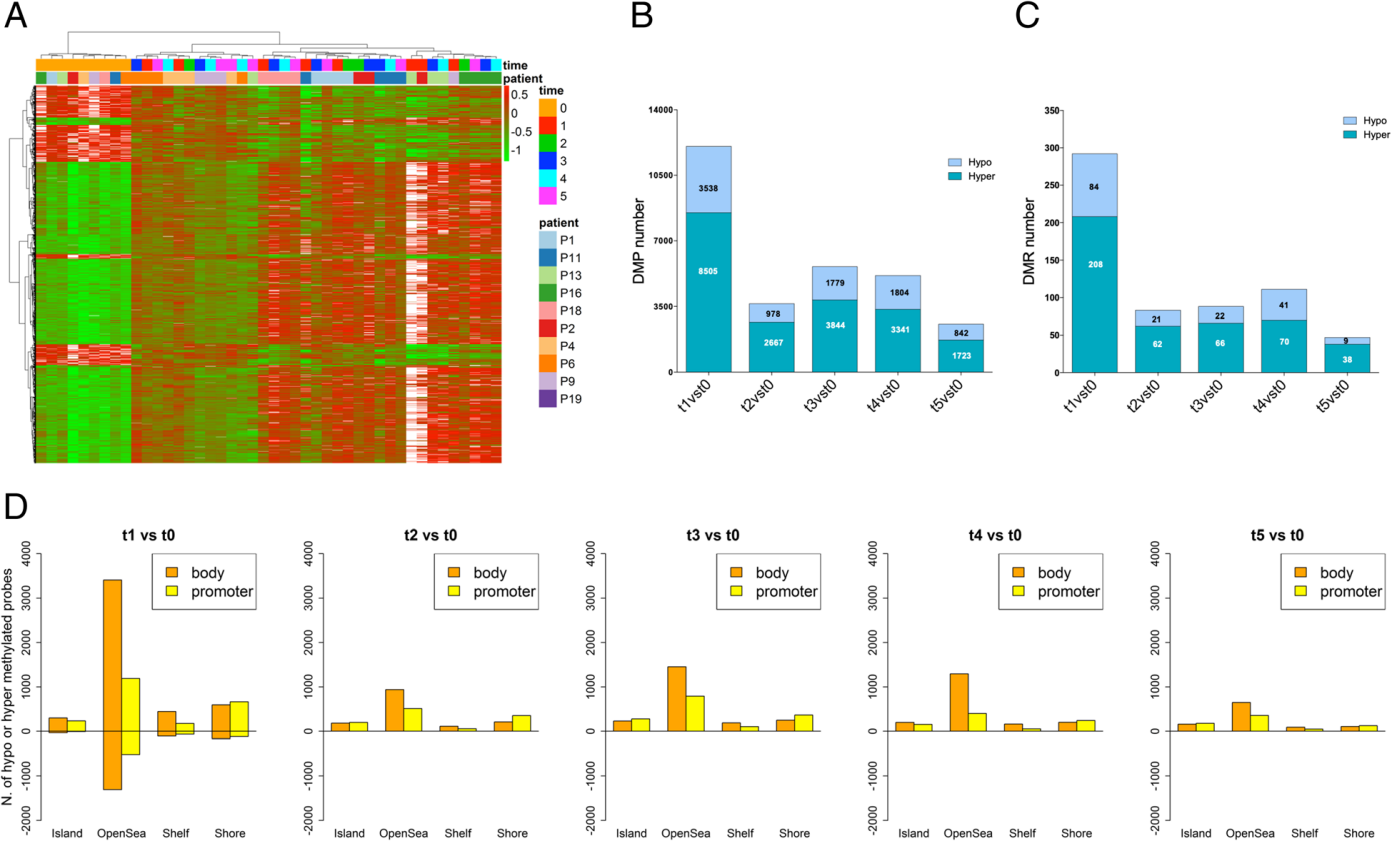
Differential methylation profile between donor and recipient HSPCs. **a** Heatmap of significant DMPs common in all comparisons between donors (t0) and recipients (t1-t5). Differential methylation was annotated by different colors (green hypo-methylation and red hyper-methylation, respectively). In the lower part of the heatmap, the numbers indicated the different time points. **b** Number of DMPs in all recipient time points (t1-t5) vs donors (t0) distinct in hypo- and hyper-methylated probes. **c** Number of DMRs in all recipient time points (t1-t5) vs donors (t0) distinct in hypo- and hyper-methylated regions. **d** Distribution of hyper- and hypo-methylated probes across CpG sites (Island, Open Sea, Shelf, and Shore) in promoter and body regions in all time points compared to t0. In the vertical bar are reported the number of hyper-methylated (0 to 4000) and hypo-methylated (0 to − 2000) DMPs

To assess potential methylation changes after AHSCT, we first clustered differentially methylated probes (DMPs) common to all comparisons between donors (t0) and recipients (t1-t5) and then we analyzed the number of significant DMPs in each comparison. The hierarchical clustering showed a different methylation profile between donors and recipients, with a prevalent hyper-methylation of HSPCs after transplant in recipients (Fig. 3a). Moreover, the analysis of DMP number identified a major difference of DMPs (*n* = 12,043) in t1 vs t0 with a marked reduction in the other comparisons, reaching 2565 DMPs in t5 vs t0 (Fig. 3b). In particular, we found a prevalent hyper-methylation of DMPs in each time point vs t0 (Fig. 3b). Similarly to the DMPs, we reported that the DMR number (containing single or cluster of DMPs) was higher (*n* = 292) in t1 vs t0 with respect to other comparisons reaching only 47 DMRs in t5 vs t0 (Fig. 3c). Also for DMRs, a major hyper-methylation was found for each time point vs t0 (Fig. 3c). These data indicated that HSPCs were hyper-methylated at 30 days (t1) post-transplant and their methylation is strongly reduced up to 365 days after AHSCT (t2-t5).

To investigate the methylation changes during AHSCT, we analyzed the distribution of DMPs across CpG sites (CpG island, shelves, shores, and open sea) in promoter and body regions between donors (t0) and recipients (t1-t5). In particular, CpG island, shelves, shores, and open sea were more hyper- than hypo-methylated in both regions (Fig. 3d). Of note, in the promoter region, open seas and shores resulted consistently more differentially methylated compared to islands and shelves in all comparisons. In the body, instead, only open seas were found more differentially methylated with respect to other regions (Fig. 3d).

### Identification of gene signature of HSPCs after transplant

To validate the methylation array data, we analyzed the global methylation level of CD34 gene in both donor (t0) and recipient HPSCs (t1-t5). In particular, two hypo-methylated regions located one in canonical CD34 promoter and another one mapping on 5′UTR of a CD34 transcript variant (ENST00000367036.7) were found in all time points. Moreover, we also observed a higher methylated region in CD34 gene body (Additional file 1: Figure S1). Therefore, expression of CD34 antigen was associated to its promoter hypo-methylation.

**Fig. 4:**
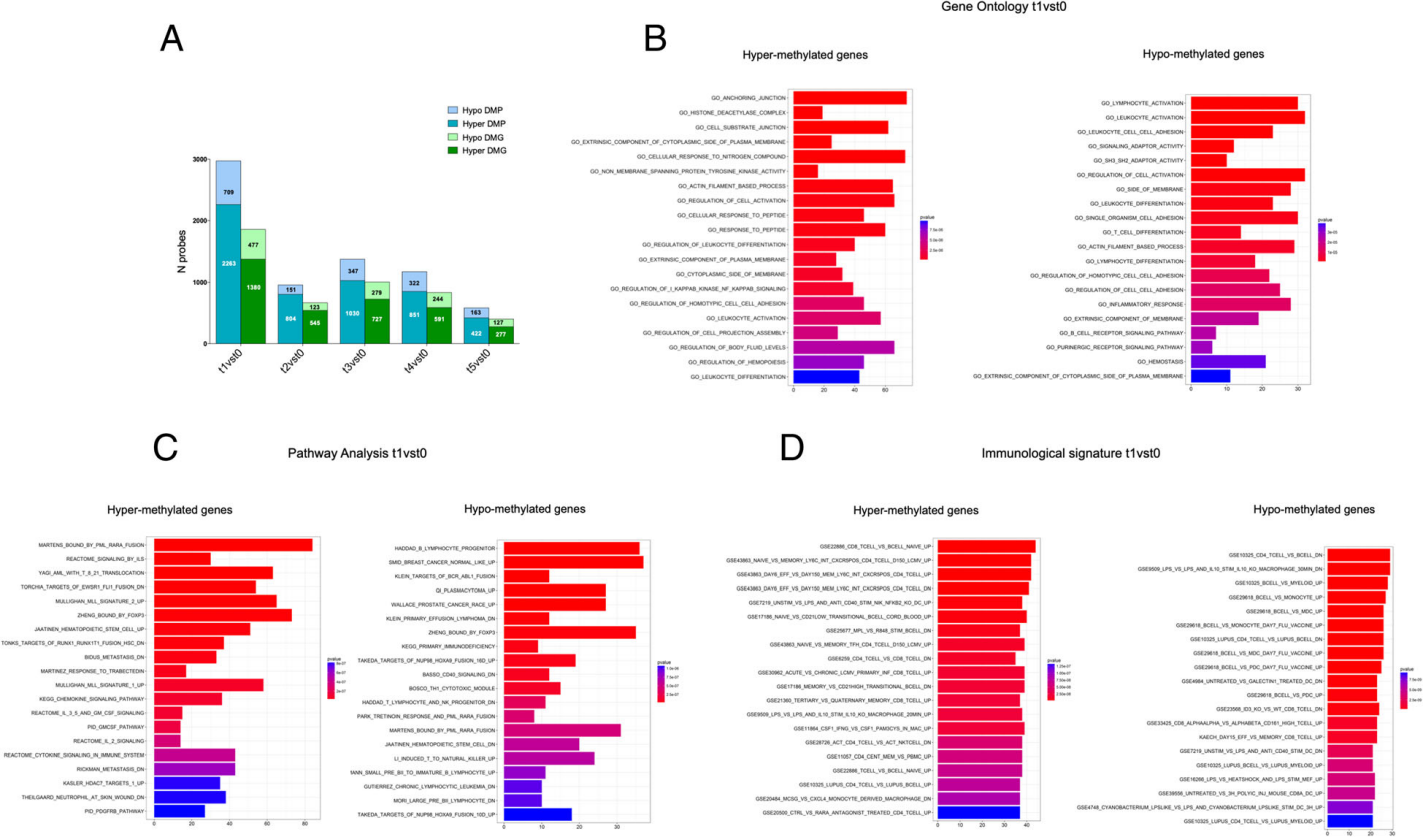
Gene signature after transplant. **a** Number of DMPs located in promoter and respective DMGs in all recipient time points (t1-t5) vs donors (t0). **b** Gene ontology analysis of hyper- and hypo-methylated genes in t1 vs t0 comparison. **c** Pathway analysis of hyper- and hypo-methylated genes in t1 vs t0 comparison. **d** Immunological signature analysis of hyper- and hypo-methylated genes in t1 vs t0 comparison

To identify genes that are modulated after AHSCT, we analyzed the DMPs localized in the promoter region, whose methylation regulated the gene expression. In particular, we compared the promoter-associated DMPs of recipients (t1-t5) with those of donors (t0). The major differences of DMP number in promoters were observed in t1 vs t0 obtaining a list of 2263 hyper- and 709 hypo-methylated probes corresponding to 1380 and 477 genes, respectively. These last numbers were drastically reduced, with a slight perturbation during 120–180 days, reaching 422 hyper- and 163 hypo-methylated probes (277 and 127 genes, respectively) in t5 vs t0 (Fig. 4a). The gene ontology analysis of t1 vs t0 indicated that both hyper- and hypo-methylated genes were significantly involved in biological processes important for hematopoiesis such as lymphocyte activation and differentiation, dynamic shaping of cellular membranes (actin filament process), and cell movement (adhesion) (Fig. 4b). Moreover, pathway analysis revealed that these genes were significantly enriched in the following categories: hematopoietic stem cell, lymphocyte, NK progenitors and chemokine and cytokine signaling, and interleukin (IL) 2, IL3, IL5 and Granulocyte Macrophage-Colony Stimulating Factor (GM-CSF) signaling (Fig. 4c). Analyzing immunologic signature data sets, we also found that some genes were involved in B lymphocyte commitment (Fig. 4d).

**Fig. 5:**
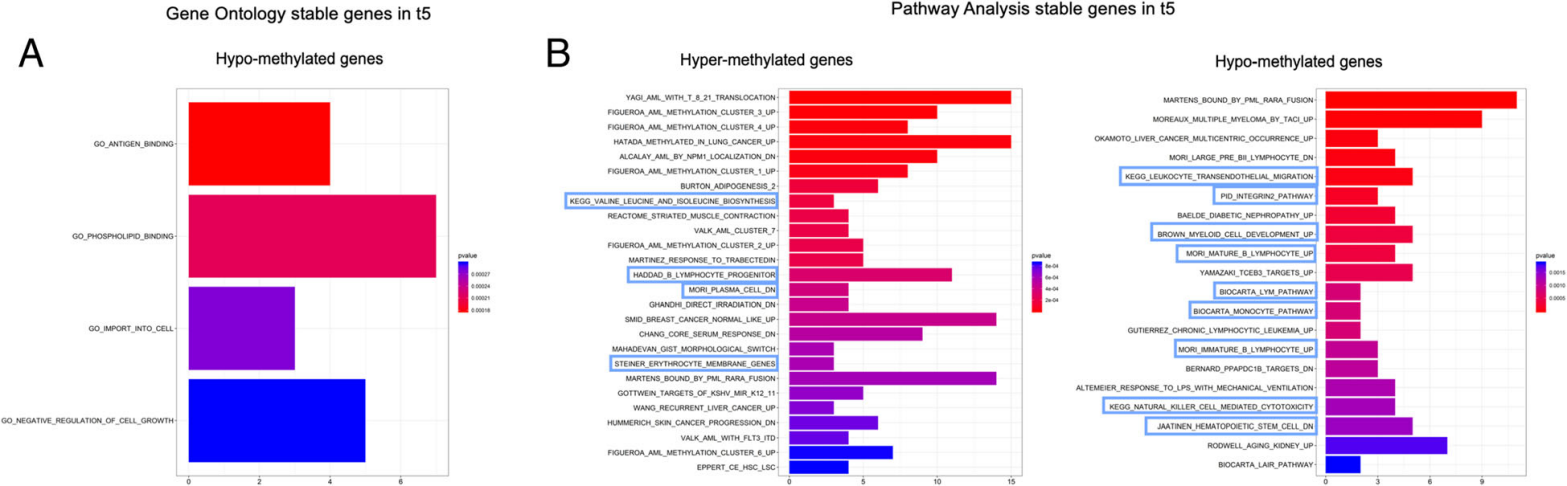
Functional analysis of “stable genes”. **a** Gene ontology analysis of hypo-methylated genes relative to t5 stable genes. **b** Pathway analysis of hyper- and hypo-methylated genes relative to t5 stable genes

To understand which genes identified at 30 days preserved methylation changes at long term (1 year) after transplant, we compared them with DMPs/genes in t5 vs t0. In particular, we observed that the majority of these DMPs/genes (2604 DMPs/1699 genes) were differentially methylated at t1 but not in t5 against t0, indicating that methylation profile after 1 year of transplant comes back similar to donors. We defined them as “revert genes” (Additional file 2: Table S1). Interestingly, we also observed that 368 DMPs corresponding to 270 genes (199 hyper- and 71 hypo-methylated genes) were differentially methylated both in t1 and t5 against t0 showing a stable modification after transplant. We defined them as “stable genes” (Additional file 3: Table S2). The gene ontology analysis indicated that hypo-methylated “stable genes” were involved in dynamic shaping of cellular membranes (such as phospholipid binding and antigen binding) (Fig. 5a). Moreover, pathway analysis revealed that hypo- and hyper-methylated “stable genes” were significantly enriched in hematopoietic stem cell trafficking, such as leukocyte transendothelial migration, integrin2 pathway, and in function related to the HSC regulation and homeostasis (Fig. 5b).

Finally, in order to understand if “stable genes” were the result of methylation changes which occurred at 30 days and if were preserved up to 1 year post AHSCT, we searched for “stable genes” in t2-t4. Of note, we did not observe change in methylation status of 270 stable genes passing from t1 to t2, t3, t4, and t5, except for 8 genes which “temporarily” modified their methylation pattern only in t2. In fact, their status resulted similar to t1 in all other time points (t3-t5) (data not shown).

### Analysis of promoter methylation profile of ALL patients with negative clinical outcome

**Fig. 6:**
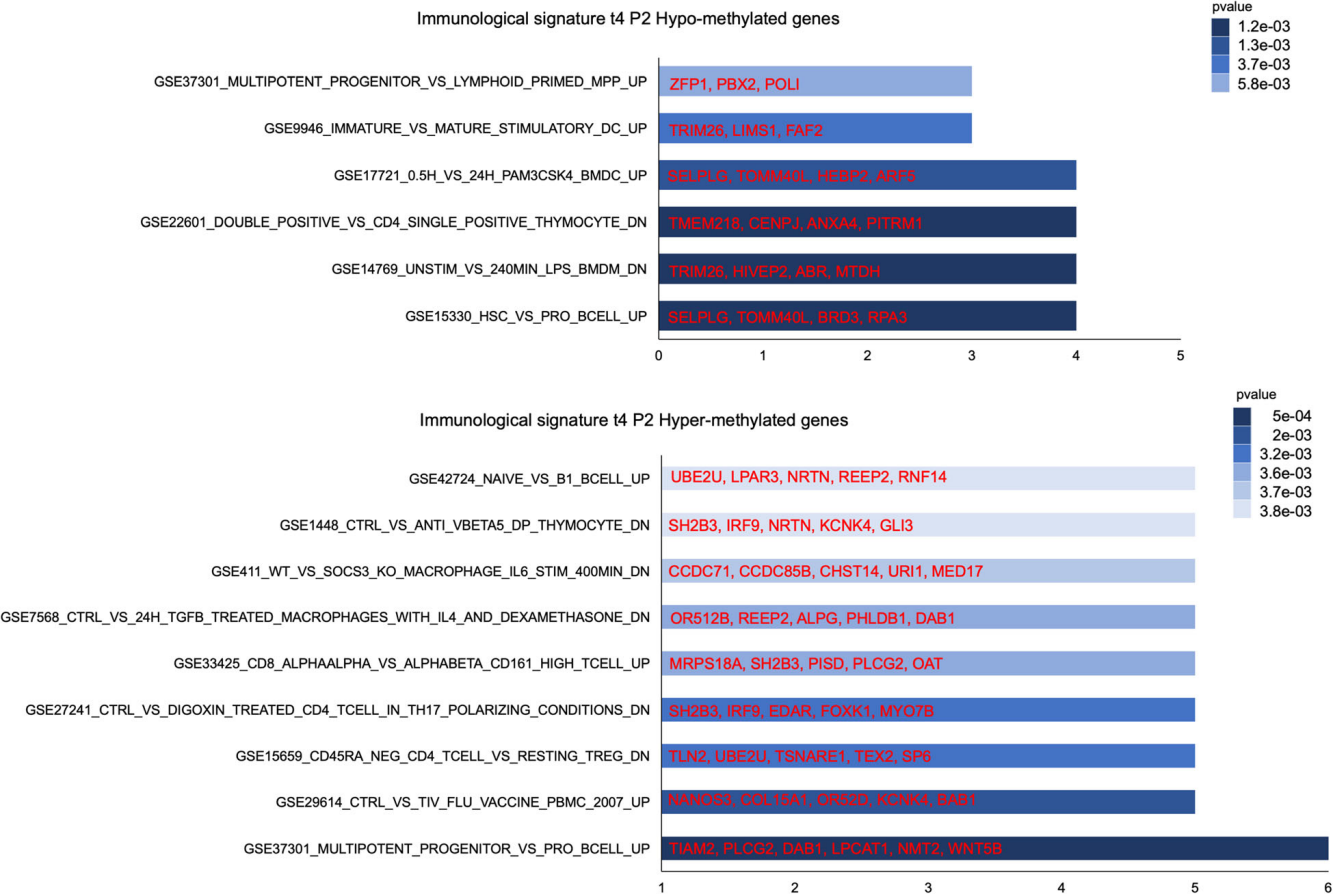
Functional analysis of genes from ALL t4 vs all other t4 patient comparisons. Immunological signature analysis of hyper- and hypo-methylated genes of P2 t4 vs t4 of all patients

In our cohort of patients, one subject (P2) with ALL relapsed and died after 195 and 300 days post AHSCT, respectively. Therefore, we analyzed the promoter-associated DMPs of this patient vs all other patients in each time point. In this comparison, a similar promoter methylation profile was found in t0-t3 (data not shown), whereas a strong difference was observed in ALL t4 vs t4 of all patients. In particular, 195 DMPs (corresponding to 143 genes) were found in t4 of ALL patients when compared to other patients (Additional file 4: Table S4). Of note, t4 methylation profile of ALL patients (P2_4) was evaluated as an outlier by MDS and for this reason it was excluded from the next analysis (Fig. 1a). The immunological signature of ALL t4 vs t4 of all patients (98 hyper- and 45 hypo-methylated genes) showed an enrichment in “hsc_vs_pro_bcell_up,” “multipotent_progenitor_vs_lymphoid_primed_mpp_up,” and “multipotent_progenitor_vs_pro_bcell_up” categories (Fig. 6).

## Discussion

The main goal of this study was to provide a general overview of DNA methylation changes in BM CD34^+^ cells derived from HM patients, including ALL, AML, and HL, after AHSCT and their relative donors. Analyzing the global methylation profile, MDS segregated the patient P19, its respective donor and t4 of patient P2 from all other samples. Interestingly, the first separation identified a strong methylation profile variation between a patient affected by HL (P19) and those with acute leukemia. This partition is probably due to the nature of two malignancies and the different conditioning regimen (Table 1). In fact, lymphoma and acute leukemia are quite different from each other for pathogenesis and clinico-pathological properties, the former starting in the immune system and affecting the lymph nodes and lymphocytes [41, 42] and the latter occurring when the BM produces too many abnormal progenitor white blood cells [43, 44]. In addition, HL patient received reduced intensity conditioning, while all the other patients were subjected to a myeloablative regimen (Table 1). Unexpectedly, MDS showed that AML and ALL methylation profiles overlap even if these are two different leukemia types, i.e., myeloid [43] and lymphoid [44] respectively. Moreover, a possible explanation of P2 t4 exclusion after MDS could be the presence of a different chimerism status (10%) with respect to the other patients (90%). It was reported that patients with mixed chimerism showed a higher differential methylation than donors, with respect to patients with complete chimerism [45]. These findings suggested that HSPC DNA methylation pattern post-transplant could be influenced by recipient BM microenvironment or by the recurrence of patient HSPCs.

Analyzing genome-wide BM HSPC methylation with or without discrimination between gene promoters and bodies, after elimination of confounding factors, we visualized a similar profile between donors and recipients during transplant at all investigated time points. These data are in line with those of Rodriguez et al. [45] showing stable global methylation levels after HSCT over 12 months between donors and recipients. However, that work had two important differences with respect to this one, the use of PB mononuclear cells and the analysis of repetitive sequences (LINE1 and NBL2) [45].

Interestingly, in unsupervised hierarchical clustering, we observed that four donors clustered together, whereas the other five were clearly separated. This distribution could be not due to the different donor types.

It was reported that HSPCs from the same source, CB or mobilized PB (mPB), of different healthy subjects all grouped together and that CB and mPB groups clustered close to each other [20, 46]. A possible explanation to our donor distribution could be their age. In fact, analyzing their characteristics, we noted that the donor cluster age ranged from 23 to 30 years, while the age of the other case was instead > 35 years and, in one case, 14 years. Moreover, four other donors clustered with their respective recipients. This cluster could be due to the nature of CD34^+^ recipient cells that maintained the “methylation memory” of donor infused cells. Indeed, DNA methylation is considered a stable epigenetic mark that can be inherited through multiple cell divisions [47, 48], but during the development and cell differentiation, it is dynamic, although some methylation changes are preserved as an epigenetic memory [47, 48]. Of note, the other half of patients did not cluster with their respective donors probably because the HSPCs were strongly influenced by BM HM recipient microenvironment [49, 50]. To be solved, this issue will need a future investigation on an increased number of subjects.

The DNA methylation profile between donors and all time points of recipients revealed a strong hyper-methylation of HSPCs after transplant. In particular, we found in all cases that hyper-methylated probes are commonly associated with CpG open seas and shores in promoter regions and with CpG open seas in body regions. Our data are in agreement with those of Weidner et al. [51] who demonstrated that cultured HPCs CD34^+^ acquired significant promoter DNA hyper-methylation in shore regions, reflected in differential gene expression and variant *DNMT3A* transcripts. Moreover, also the open sea hyper-methylation is in accordance with the methylation data observed in other studies carried out in healthy tissues, but in contrast with its hypo-methylation found in various cancers [52–54]. DNA methylation of CpG shores, shelves, and open sea has been shown to be related to gene expression in normal and tumor cells [52, 55, 56]. In particular, hyper-methylation of the gene bodies open sea was positively linked to gene expression in different human tissues, cell lines, and primary cancer cells [52, 53]. On the other hand, the hypo-methylation in promoter regions, specifically in open seas, shelves, and shores, was significantly associated to upregulation of the corresponding genes [52, 54].

Interestingly, we observed a relevant change in terms of DMP/DMR/gene number in patients after 30 days of AHSCT, with respect to the donors. These numbers drastically changed, going from 12,043/292/1857 at 30 days to 3645/83/668 at 60 days, and this reduction was maintained for up to 1 year after transplant. It was suggested that CD34^+^ donor cells consistently modified their methylation pattern during the engraftment phase, which occurs 2 to 4 weeks after transplant. This perturbation could be caused by the recipient microenvironment, which strongly influenced the CD34^+^ engraftment [57]. Recent studies identified the intimate association between BM perivascular endothelial cells and HSCs throughout stem cell life, identifying their important role in regulating HSC biology [49, 50]. In particular, different molecular and physical properties of microenvironment cells critical for HSC engraftment, maintenance, localization, and regeneration have been described [49, 50]. In addition, this perturbation was associated to a dominant hyper-methylation that was drastically reduced in time, suggesting an initial genome silencing which decreased within 60 days and then for up to 1 year. Weidner et al. [51] demonstrated that DNA methylation of healthy CD34^+^ was hardly affected by stromal support. Moreover, microenvironment has been shown to exert profound but partially reversible changes on DNA methylation and on mRNA expression profile in patient-derived glioma stem cells [58]. Functional analysis of the possible consequences of this perturbation showed its involvement in hematopoietic cell activation, differentiation, shaping, and movement. Concerning methylation restoring, it has been reported that DNA methylation is inheritable and adapts to a specific cellular memory function during development or stress [48, 59], cellular conditions that applies to this case.

It is known that the immunological reconstitution of different cell subsets after AHSCT occurs at different time points: 21 days for neutrophils, 30–100 days for NK cells, 100 days for T cells, and 1–2 years for CD19^+^ B cells [60]. Anyway, little is known about methylation levels and gene expression in the engraftment phase. Our data showed an involvement of methylation in hematopoiesis specifically in this phase; in fact, post 30 days of HSCT, the gene ontology revealed a modulation of leukocyte activation and differentiation while pathway analysis showed the regulation of lymphocyte and NK progenitors and chemokine and cytokine signaling. In particular, cytokines like IL-3 and IL-5 and growth factors including GM-CSF are involved in proliferation and differentiation of myeloid precursors [61–63]. Moreover, they also regulate HSC quiescence/self-renewal and lymphoid commitment activating signal transducer and activator of transcription 5 (STAT5) [64–66]. In conclusion, during the engraftment phase, the modulation of these pathways is required for HSC quiescence/expansion and for an efficient lympho-myeloid repopulation [67]. Moreover, an enrichment in immunological signature identified genes involved in lymphoid commitment. The lymphocyte reconstitution after HSCT has an important role, not only on the prevention of serious infections in the early transplantation period, but also on the killing of residual leukemic cells by graft-versus-leukemia effect [68, 69]. Other studies reported that a higher absolute lymphocyte count on day + 30 was associated with faster hematopoietic recovery and, consequently, a more rapid neutrophil and platelet engraftment [68, 70]. Of note, it is intriguing that at 30 days post-transplant in PB there are circulating leucocytes indicating an occurred engraftment, while in BM their progenitor cells are still subject to a strong perturbation of methylation status.

Our analysis proposes that it could be interesting and useful to anticipate the methylation analysis of CD34 before 30 days. In fact, a CD34 methylation pattern at 10–14 days after AHSCT could point out all the changes acquired from the CD34 in the “full” engraftment phase. We could define an “engraftment methylation signature” of CD34 thus using it as first sign of engraftment compared to both white blood cell and platelet count. Indeed, it could allow an early planning of supportive therapies in transplantation.

Remarkably, at 60 days post HSCT, CD34^+^ cells mainly re-established the same gene methylation levels as donor HSPCs, except for a gene pool that remained differently methylated up to 365 days. These genes encoding for matricellular proteins, *α*/*β* integrins, and chemokines are involved in dynamic shaping of cellular membranes and trafficking. Chemokines are a small group of related chemoattractant peptides that play an essential role in the development and homeostatic maintenance of the immune system [71, 72]. In particular, they regulated HSC homing to their BM niches and directed immature lymphocytes to a series of maturation sites within lymphoid organs [71, 72]. Like chemokines, also the *α*/*β* integrins, a class of heterodimeric trans-membrane receptors, play an important role in HSC maintenance, regulating their egress from the BM niche and other functions [73]. Within the BM niche exists a tightly controlled local microenvironment that regulates quiescence, proliferation, and differentiation of HSCs, in order to ensure life-long, balanced, and multilineage hematopoiesis [49, 74]. Overall, our data suggest that BM recipient microenvironment regulated/modified several cellular responses of CD34^+^ cells, allowing their adaptation to novel environmental stimuli.

To verify the presence of any relationship between the outcome after transplant and the donor type, we compared the methylation profiles of patients related to those with unrelated donors. In particular, no differences were found, except in t4 in which two differently methylated genes (*CMYA5* and *ZNF432*) were identified (data not shown).

Finally, the promoter methylation profile analysis at 180 days after transplant of ALL patient showed a strong difference with respect to other patients. We observed a modulation of methylation pattern regarding B and T cell populations. The immunological signature enrichment revealed the presence of genes, such as *BRD3*, *PBX2*, and *WNT5B*, involved in HSPC proliferation, self-renewal, and differentiation, found deregulated in leukemia [75–78]. In addition, among deregulated genes, we found GLI3, a negative regulator of the Hedgehog signaling pathway, which is aberrantly activated in cancer. In line with our data, GLI3 was demonstrated to be epigenetically silenced in patients with acute leukemia [79]. Interestingly, at this time point, ALL patient had 10% of chimerism and after additional 15 days relapsed. This suggests the interesting hypothesis that promoter methylation profile analysis may be useful to predict relapse in these cases, although further investigation is needed to validate this possibility. In line with our hypothesis, a recent study investigated the prognostic relevance of CpG island methylation phenotype classification in pediatric B cell precursor (BCP)-ALL patients, showing that it is a strong candidate for improved risk stratification of relapsed BCP-ALL [80]. We envision that a CD34 promoter methylation pattern study could integrate the routine diagnostic tool of chimerism analysis in assessment of engraftment and early detection of graft failure in AHSCT.

## Conclusions

This study analyzed, for the first time, DNA methylation dynamics of BM-HSPCs after AHSCT which involved multiple adaptation steps in the new recipients. Methylation consistently changed at 30 day post AHSCT and progressively returned to levels similar to those of donor HSPCs. An acquired modification of DNA methylation persisted, however, in only a pool of genes for up to 1 year. Finally, preliminary data in one ALL patients suggested that methylation profile analysis could provide a predictor of relapse.

Despite the relative small sample number, our data strongly suggest that DNA methylation analysis is a valid complement to studies on AHSCT based on gene expression profiles, providing also useful information for further investigation to characterize the epigenetic mechanisms occurring in transplants. It can be reasonably expected, in fact, that detailed DNA methylation analysis, as a source of novel biomarkers, will help advance toward precision medicine in AHSCT and hematological malignancies.

## Additional files

Additional file 1: **Figure S1.** DNA methylation in the genomic region mapping on CD34 gene in donors (to) and in receiving time points (t1, t2, t3, t4, t5). (DOCX 652 kb)

Additional file 2: **Table S1.** Hypo- and Hyper-methylated “revert genes”. (DOC 86 kb)

Additional file 3: **Table S2.** Hypo- and Hyper-methylated “stable genes”. (DOC 36 kb)

Additional file 4: **Table S3.** Hypo- and Hyper-methylated genes deriving from p2 t4 vs all other t4 patients comparisons. (DOC 33 kb)

## Abbreviations

AHSCT: Allogeneic hematopoietic stem cell transplantation
ALL: Acute lymphoid leukemia
AML: Acute myeloid leukemia
BM: Bone marrow
CpGs: Cytosines adjacent to guanines
DMPs: Differentially methylated probes
DMRs: Differentially methylated regions
HL: Hodgkin lymphoma
HLA: Human leukocyte antigen
HMs: Hematological malignancies
HSPCs: Hematopoietic stem and progenitor cells
MDS: Multidimensional scaling
mPB: Mobilized peripheral blood
P: Patient
PB: Peripheral blood
TSS: Transcription start site
UCB: Umbilical cord blood
UTR: Untranslated region
