# Supplementary material for: DNA methylation dynamic of bone marrow hematopoietic stem cells after allogeneic transplantation
Source: Stem Cell Res Ther. 2019 May 20;10:138. doi: 10.1186/s13287-019-1245-6 (PMC6528331; doi:10.1186/s13287-019-1245-6)
Supplement: Supplementary file 1 — Figure S1. DNA methylation in the genomic region mapping on CD34 gene in donors (to) and in receiving time points (t1, t2, t3, t4, t5). (DOCX 652 kb) [file 13287_2019_1245_MOESM1_ESM.docx]

**
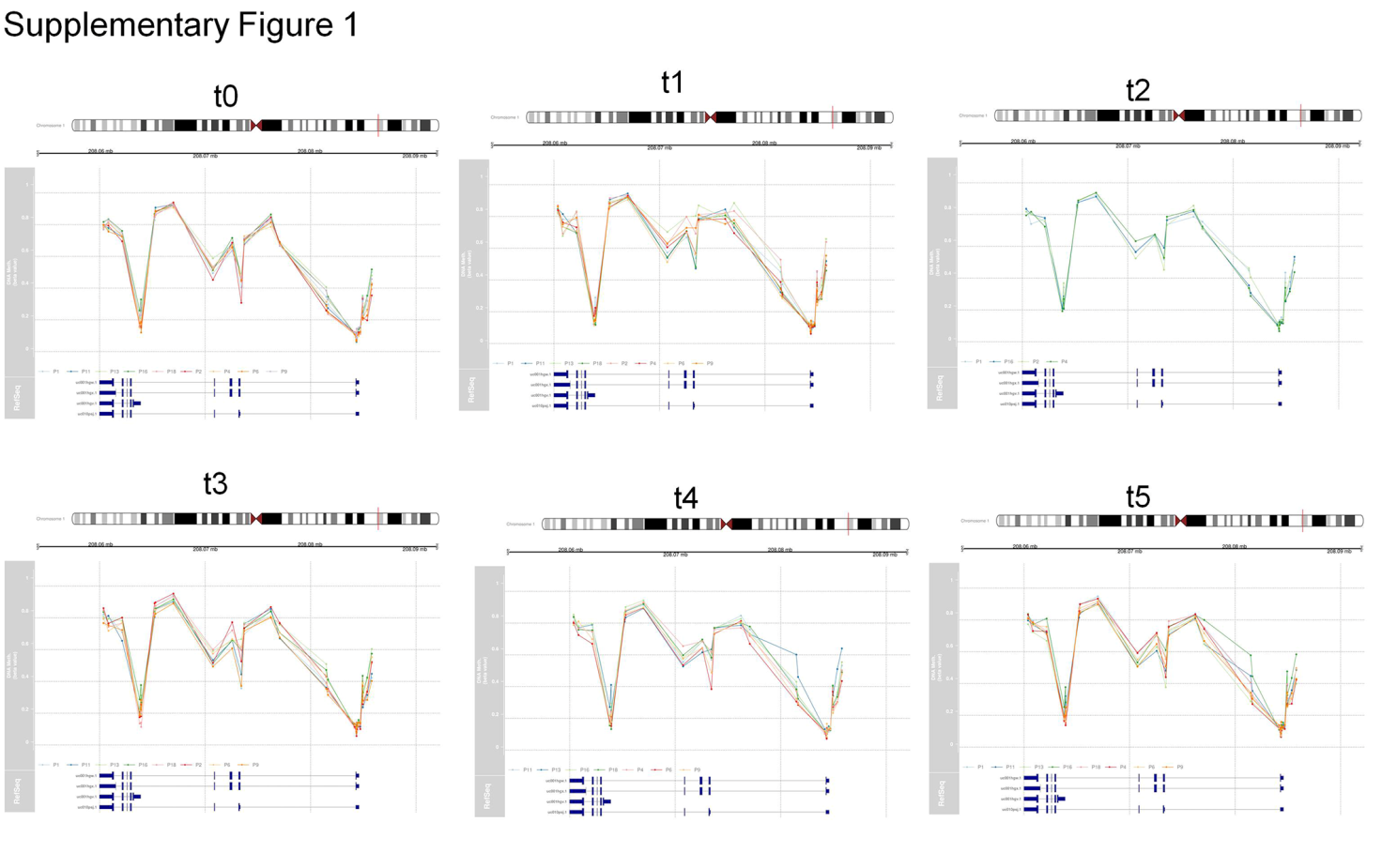
**

**Additional file 1: Figure S1.** DNA methylation in the genomic region mapping on CD34 gene in donors (to) and in receiving time points (t1, t2, t3, t4, t5).
