# Supplementary material for: DNA methylation dynamic of bone marrow hematopoietic stem cells after allogeneic transplantation
Source: Stem Cell Res Ther. 2019 May 20;10:138. doi: 10.1186/s13287-019-1245-6 (PMC6528331; doi:10.1186/s13287-019-1245-6)
Supplement: Supplementary file 2 — Table S1. Hypo- and Hyper-methylated “revert genes”. (DOC 86 kb) [file 13287_2019_1245_MOESM2_ESM.doc]

**Additional file 2:** **Table S1.** Hypo- and Hyper-methylated “revert genes”.

| Hypo-methylated revert genes | Hyper-methylated revert genes |
| --- | --- |
| RAG1  LTA4H  A2M  FAM49A  IRF1  CYFIP2  SPATS2L  SH3BP2  FAM71B  PCED1B  ST18  BCAR3  ASAP1  OR5A2  KSR1  MGAT4C  NAPSB  SEPT9  GNG7  FAM129C  LIMK1  EHD4-AS1  POU2AF1  FBXO32  LOC101929574  SH2D4B  GRB10  TRIM26  P2RX1  ADGRG7  TNF  ARHGEF3  KLHL30  ADGRE1  PTCRA  FAM111B  CLNK  CD19  CALCR  SLC2A5  GPRIN2  PKIG  VPREB1  MYLK  AOX1  CDKN1A  MIR8064  MIR339  PPFIA4  NT5C  THEMIS  INSC  SERPINA1  GBP5  LY86  TLR6  MIR3945  ADORA2A  SVIL  PATL2  LTA  LILRB4  MAP3K7CL  RAD54L  SEMA5B  LRRC31  FANCC  KIAA1199  LOC283710  SRBD1  CCDC162  C16orf74  PLEK  PDE9A  MIR496  IL10  GATSL3  CDYL  CLIP2  ZHX2  SLC14A2  ITPKB  C10orf28  P2RY14  NAA30  PIK3R5  TBC1D16  CD86  CD22  LOC151484  POPDC2  C17orf88  BLK  DNMBP  LPP  CD96  GPR128  TMEM63B  GP9  GNB1  CCR6  IL7R  ASB10  CD38  CEP70  FGD2  CTCF  IFITM1  ACSL3  CBFA2T3  SORBS3  PRKAR1A  FBXW7  TRAK1  NCK2  WDFY4  GNGT2  ABI3  C11orf74  RAG2  LOC100506801  EEF2K  SPATA32  LCK  DNAH17  FRYL  HTR2A  TTLL10  FGD4  RPA3  UMAD1  LINC01046  ARHGEF7  KIAA1409  ITGAD  HOXB4  GPR65  WDR49  LPAR5  KIAA1949  LY9  MIR194-2  MIR192  GIMAP8  GPR18  NCR3  PER2  KIAA0513  ABR  PLEKHG3  PLEKHG1  SIPA1  TCF4  CORO2B  MS4A1  MIR429  PLSCR1  MOB2  NOX3  EPB41L1  P11  ANGPTL1  LINGO4  NEK6  BLNK  S100A3  C18orf1  NEU4  SIT1  CDC42EP3  TMEM51  FOXP1  LDLRAD3  BBX  KLHDC7B  ZMIZ1  ZNF233  GJA5  GALNT14  OR2L13  HTR3A  C20orf196  SH2D3C  MIR548W  BCL2L14  PC  LINC00544  LSP1  SEMA6A  HHLA2  MYL4  GPR87  TRPM2  WASF2  SYK  SMIM14  FCER2  ANXA4  MB  TPX2  BAHD1  CD79A  LOC101927189  CD53  TNFRSF13B  HHIP  RNASEH2B  ESM1  UNC79  HMCES  AKAP13  CNTN5  AMZ1  MTA3  JRK  APBB2  N4BP3  PLXNB2  ZFP2  MS4A7  TRPV4  MIR4740  CEP85L  NUGGC  MAP1LC3B2  GLRX  RFX2  FXYD2  VGLL4  GPR171  CCDC70  LGMN  LCN6  MPO  LSMEM2  SGMS1  SPATC1  LY6E  RNF19A  CTNNA2  PRF1  TRERF1  MIR4442  GAS2L3  SGSM3  ALPL  HIVEP3  SEMA4D  RNF165  CLEC2D  BAHCC1  LINC01447  C6orf59  RELT  RAD52  OR6P1  CERS4  TNNI1  CLDN14  NRXN1  CREM  CECR3  PLEKHB1  DPF3  CHST15  C5orf20  NPFFR1  PAK4  C9orf139  FUT7  A4GALT  RBMS3  PMEPA1  SCML4  PAG1  HSD11B1  USP18  SHANK1  ATP2B2  PIK3CA  TCAF1  CD72  ST6GAL1  JAK1  ASH1L  MDM4  CD36  LINC01507  FGGY  IL15  GPER  SLC37A3  ANKRD22  SP110  NAE1  ADGRG1  SLC38A1  EIF4E3  CLIP1  NCRNA00114  SLC22A18  ZNF721  AMBRA1  EDN2  ITGBL1  NOD1  PAPD7  LRRC8D  FAIM3  OSBPL3  GUK1  MACC1  BACH2  DUSP27  RERE  FSD2  KLRF2  EPN2-AS1  EPN2  RNF43  PRKD2  WARS  WDR25  DTNA  FBXO5  ZNF385A  MGAT1  RAPGEF1  TLR9  CXCR5  TIFAB  TNK2  NT5C2  LZTFL1  IGLL1  SUV420H1  GPR151  EIF3G  DCUN1D3  CD180  KCNJ13  PLEKHF1  UMODL1  MIR3150B  ZNF146  SLCO2B1  SLC4A8  ZNF710  CD28  ARID5B  HIVEP2  ATXN1  HSH2D  CREB5  PIK3C2B  ARMC3  CMTM5  RGL4  SLC23A1  DNAJA4  CYTIP  MICAL2  P2RY6  IL18RAP  ELFN2  ARHGAP27  RUFY3  SLC17A3  RARG  PRKCH  BANK1  CD1C  BIK  HOXC4  SMARCA4  NEK10  KCNJ15  MX1  KHNYN  DNAJC5B  C10orf54  GJC1  WIBG  GSN  PACSIN2  KIR3DX1  ST3GAL1  PRTN3  LOC439933  PRKCZ  LOC440028  ST5  MTUS1  GFY  PRDM11  STARD13  MIR634  RASGEF1A  L3MBTL4  JCHAIN  MAP7  PLA2G5  NDUFAF3  DALRD3  MIR425  ADPRHL1  TMPRSS11GP  GTDC1  CLEC12A  FHIT  MYH9  GNG4  ART3  LAMB3  MYT1L  ZNF608  MBP  IFI30  RIMS3  RASSF4  SYT1  RHBDF2  CPEB3  C12orf62  SOX18  SLC29A1  MEF2D  TNS3  DSE  TMEM71  PLEKHA6  SLIT1-AS1  TNS1 | OXR1  TANK  SOCS5  CDH5  CMTM2  B3GALT4  ANKAR  SLC25A25  SPINK4  RUFY1  RHOT2  CASC4  C1orf122  ATP2B4  SIPA1L3  UBASH3A  C1orf186  RERE  PRR5L  TMEM14A  ARHGEF18  AP2M1  C5AR2  C6orf27  CTNND1  ADAM8  FAM196B  TESPA1  PRDM11  TTC39C  DPPA4  ICAM4  MIR126  LIMK2  SGK494  NR1H3  CLASP1  C10orf71  MTHFR  REM2  PCGF3  ELF1  TBL1XR1  LZTFL1  H2AFY  GAB2  ETS1  HDLBP  KIAA0182  EFNB3  CABLES1  HRASLS5  IL3  FBXO22  SMCR5  RAI1  TNK2  GNB5  GNAS  VNN1  SLC23A2  HNRNPM  NRIP2  ADCY4  HECTD4  QRFP  CSNK1G2  MLKL  PROM1  ELAVL1  ACCN3  TRPC4AP  HIC1  LCN6  MAP3K13  CCNY  CRYM  SIN3A  GATAD2A  SQSTM1  SLC6A6  LOC728743  AREL1  PACSIN2  UNKL  BTBD3  USP21  VGLL4  WFIKKN1  SP7  BACH2  HIVEP3  CAP1  IFFO1  RAP1B  SSBP4  RHOH  EXD3  STAP2  SETD5  ZMIZ1  TPI1  NACC2  EIF2AK2  JADE1  GP1BA  ARAP1  TRERF1  CELF1  GUSB  NOD1  LRRC73  AMAC1L3  ZBTB4  ZBTB7B  LCP1  MSRB3  ABHD16B  CCR4  CDC42SE1  ZNF395  TREX1  NCOR2  ITGAX  MAP1LC3A  RAB32  POGZ  SYNGR1  ABHD14B  ABHD14A  HSD3B7  PTH2R  EPB49  LRRC29  CCDC57  LPIN1  FAM43A  BANP  MAFG  SNORD127  RBM34  ZNF48  SFXN2  TMEM204  ALOX15B  RSPH6A  PLCB2  CCDC75  ANKRD52  QSER1  SIL1  ZFHX3  LIMS1  CHST12  PITPNM2  C2orf67  SH2D3C  CSF3R  PRDM8  FAM167A  ADGRG1  ZC3H7A  DOCK1  CCND3  CBFA2T3  PNN  SETD6  CSF2RB  LINC01229  TMEM40  SLC10A5  SH3GLB1  KRT1  C16orf5  BEND7  CPNE2  LAG3  C1orf216  KCTD13  OSBPL6  PRRC1  SNORA8  SNORA18  SNORD5  ZMYND8  TCN2  PES1  B3GALNT1  STAB1  EIF4G1  NACC1  LTBP3  FYN  TGFBRAP1  MYCT1  LYN  CORO1C  RELT  OSER1  FRYL  NCKAP5L  ACOT11  PLXNB1  MBD5  BRWD1  BRWD1-AS1  KATNB1  DYRK1A  MIR589  TPD52L1  C10orf76  ITIH1  TAF4  IQSEC3  TNS3  GRSF1  HSH2D  DDHD1  KIAA0125  DCI  INHBA-AS1  DNAJC4  ARHGAP27  SRC  WDFY4  JAK3  TMEM209  BID  SLC19A1  LOC101929331  MADD  EVA1B  B3GALT2  TCTEX1D4  BTBD19  PRR5  PRR5-ARHGAP8  SLC35C2  PTPRA  XPO7  GNA12  CHEK1  URGCP  GNG7  KIAA0430  NDE1  HOXA6  OPRL1  C20orf201  FAM113B  ZNF486  RAP1A  ANKRD12  PDF  NKX2-3  EXOSC10  NGEF  ANXA6  HMHA1  LRRC8B  ADGRE5  ARHGEF7  RASGRP3  ZBTB17  OTUD6B  CSNK2A1  KRT80  ZNF592  ELMSAN1  SH2B2  JAK1  PYROXD2  ITPRIP  MIR5094  RRBP1  C8orf73  LINC01301  GAPVD1  GHRLOS  TMEM184B  HTR2B  ZBTB18  ADCY7  BLVRA  C21orf58  GATAD2B  MFSD6  CYB5R3  C3orf67  TNKS1BP1  CUL1  THRA  SLAMF8  PIK3CD  SGSM3  MAP1S  TMEM125  NKIRAS2  AQP1  EZH2  ERMAP  RFTN1  TNFRSF6B  NAA60  TAPBP  C6orf89  KDM4B  CTTNBP2NL  TRAF1  FLI1  LOC100652999  AVP  PTEN  HECW2  NCALD  GSN  CLCN6  IL27RA  GPR182  GNG12  CMIP  BAZ2B  AIFM2  PPP1R10  NCOA2  LIMD2  NUP93  YWHAZ  DDHD2  C22orf26  ADORA2A  SHC1  GFI1B  C15orf52  SOX13  TMBIM1  CD2BP2  SUMF2  MYH9  CHD4  KCTD11  PVT1  NCOA7  NCOA7-AS1  TNIP1  PCED1B  AGPAT3  C1orf56  TRIP12  INSIG2  PAPD7  SLC25A34  ULK3  HPCAL1  FUT6  GIT1  EXOC3  NPPA  MIR1205  LBX2  HIVEP2  CHRM4  CNR2  CEP83  CTBP2  PRKAG1  PTGER2  UCP2  C21orf96  EPHX1  UQCR11  SUN1  ST7OT2  ZNF384  CTNNB1  PIK3R6  ZNF175  NAT15  KHNYN  CBLN3  GPR52  B4GALT1  JMJD8  ICA1  FBXO34  CSRP3  PEX11A  WDR93  PRRT2  TAOK3  ADAR  PTGDS  SLC25A20  CXCR6  RIN1  RUVBL1-AS1  PLXNC1  FGD3  HMOX2  NMRAL1  CTF1  ACY1  ADAT3  SCAMP4  CARNS1  OGDH  FDXR  SLC2A4RG  PNISR  STAT3  KHK  CXCL10  ART3  ZDHHC1  HPS4  SRRD  SNORA11B  C14orf159  ZMIZ2  ST6GAL1  RD3  SMAD3  PRR34  LOC101929633  C20orf117  SET  CRHBP  DHX32  GYLTL1B  STARD13  SHF  TLR10  SLC37A4  MAPK12  ATP6V1A  PREPL  HNRNPC  GTF2IRD1  ARF1  GPR21  C19orf24  NDST1  SPATS2  FHL2  SEC14L1  D2HGDH  TRAK2  SOX15  FAM65A  HOXA3  SH3BP4  CFLAR  TCP11L2  DOK2  SNORA68  CENPM  TSPAN4  MIR1914  MLLT11  H1F0  RHOG  SEMA4C  LYSMD2  C3orf62  VWA1  CUEDC1  PSMA6  FDFT1  LRRC33  B4GALT4  SNAP23  CASP8  NADK  ZBTB7A  APOB48R  SEPN1  B3GNT8  MICALL2  TP53I11  CLDN6  KIFC3  ARHGAP26-AS1  PLCH1  BEND3  SGMS1  MBP  ITPR1  GNRH2  GPR137  BAD  ANKMY1  LPIN2  MYLK3  TMEM91  DGKA  MPHOSPH9  MUM1  KCNJ15  C22orf32  GATA2  HTRA4  PGAP1  ANKRD2  LMO2  TPST2  FOXP1  UCK1  TIGD2  TLR6  PCYT1A  AGO3  NOSIP  FAM83A  TNFAIP3  AMIGO3  NIN  CEND1  STAT5A  RARA  NRROS  RAB27A  IPO11  MECOM  SH3BGRL3  C12orf10  RASSF9  TBC1D16  C5orf24  UBE2I  ISG20  NIPAL3  NDFIP1  STK40  C3orf27  NOD2  SCO2  PDGFD  CLDN15  SNORD126  CCNB1IP1  SSH3  RNF165  ANKRD11  AOC2  TRIM26  SIGIRR  KSR1  RBM15B  C19orf23  SMARCA2  TPM4  MRPL28  DYRK1B  ZMYND17  MVP  CDC42EP3  PSD4  LINC01176  SPHK2  PARVG  MAS1L  GPR56  PNMA1  PPAN-P2RY11  PPAN  CD82  KLHL21  PLVAP  RORC  RASA2  OTUD4  MMP14  SLCO4A1  MBNL1  STK19  LINC00332  SDK1  SDCBP2  GPD2  NFE2  DNAJB6  MGST2  TIPARP  SH3GL3  PSTK  VARS2  LRRC8D  CACNB3  SLC25A30  UEVLD  GNB1  C20orf135  ZCCHC8  THRAP3  MAPK1  ST7-AS2  DNASE1L2  LRRC8A  GPR183  FNBP1  NOL8  CENPP  HLA-DMA  TOM1  HMG20A  C1orf21  ELMO1  PLEKHA6  AKR1A1  ORAI3  TEK  C3orf42  PAK4  ARPC1B  GABARAP  BGLAP  PRPSAP1  TAF1C  EPGN  AMPD3  BCL6  SKP2  DPEP2  MAP3K14  IL4R  DCP1B  MINOS1-NBL1  NBL1  SULT1C4  UNC84A  C5orf4  ICAM2  PCBP2  RREB1  NDUFA8  MORN5  ITGA2B  CLEC3B  WHSC1  RAG2  C11orf74  PLEKHN1  SLC37A1  MAFK  TMEM150B  SMAD1  PPARG  CASS4  LINC00898  CREB1  SYK  CFL2  C10orf27  RALGPS2  PPP2R5B  FAM107B  DTX1  ACTG2  REN  AFF1  TCEA2  FGFBP2  KLHDC5  GPR110  SORL1  PRUNE  ZFP36L1  TMEM216  DNAJB14  ESYT1  FBXO18  SENCR  THAP4  RNF103-CHMP3  PLEKHG3  TTLL4  FMN1  WDR53  NFAT5  FGD4  SH2D7  MIR1203  SH3BP5  CMTM1  CARD8  PRKG2  ATP2C1  RNF2  NADK2  MIR3122  PPFIBP1  TACC1  FXYD3  BLCAP  PLEKHB2  EFNA4  HEPACAM2  TSPAN32  IP6K1  CLUAP1  PKIG  STXBP3  ST3GAL1  INF2  RALY  MST1R  CABC1  TRAF5  MKNK2  MACC1  LINGO3  GAS7  ZBTB46  ANXA11  PDLIM1  HCG22  SEPT9  AHCYL2  SPECC1  PTPRCAP  C10orf58  PPM1B  EGF  E2F5  C16orf58  NCR1  CPNE3  FAM219B  GRB2  CLIP1  GTF3C2  RGS6  RALB  EEF1D  PTPN7  RHOF  ROBO4  FAM53A  SUSD6  MEPCE  AHDC1  ZBTB38  KRCC1  PHGR1  PDE4D  SPOPL  AMD1  KDM2A  IRF5  PRKAR1B  CDC42SE2  CNPY3  FSBP  RAD54B  CNST  ZBTB2  RAPGEFL1  GPR44  STX5  NEDD4  PANO1  SLC25A22  COASY  PPP6R1  CEP295NL  SNX10  MIRLET7A3  MIRLET7B  C15orf53  SEL1L3  FER1L6  ZNF710  NUCB2  TP53INP1  CPA3  PLA2G6  CKAP5  GTF2H1  FBXL14  NFKB1  C20orf141  PPP1R16B  TASP1  UMODL1  FAM65B  OXT  KIAA1257  SCMH1  PER1  MAPRE3  OXER1  MIR3614  MIR600  SNAR-E  SEMA5A  NOP2  BCLAF1  CANT1  MAP1A  TM4SF1  TM4SF1-AS1  ZNF462  HOXA5  C9orf78  USP20  TMEM149  CHRM3  NEK6  GCNT2  LHFPL2  MAP2K1  FRY-AS1  CALN1  HERC1  NEK7  HADH  LDLRAD4  ADAMTS10  NUDC  FASTK  ERCC6  TNFAIP8L2-SCNM1  TNFAIP8L2  IKBKB  DAAM2  SGPL1  CEP68  C9orf103  ADRBK1  TNFAIP8  SCOC  SUSD3  LOC100129940  DCTN1  BAZ2A  ZDHHC7  MFAP2  ANKRD27  C1orf100  RNF145  MERTK  TLE3  AKAP13  ZNF358  TRIM69  RAB7A  NECAB3  SOX12  FAM176B  FAM165B  TXNDC11  MOBKL2A  LPAR2  FER  FCHO1  RILP  IDO1  TICAM2  ACAA1  MYD88  MIR6838  BTN3A2  ZNF697  SPECC1L  ADPRH  GAPT  PGM1  PLXNB2  TAGLN2  C15orf54  SLC14A2  PHLDB3  NCK2  POLS  CYB561  EGFL7  DIDO1  ZNF521  ALDOC  TMEM132A  KLC1  MPP7  STAT5B  PRKAR1A  CAPN14  SPDEF  SLC35A3  PPBPL1  NME4  CLASP2  C12orf65  KIAA0513  CERK  PACS2  HMBS  HHAT  UTP11L  SLC39A13  SLC27A3  DNAJB13  FAHD1  ZBTB20  ACVR1  PLD6  STAMBPL1  CSRP1  PSMG2  SPG7  MAP3K7IP2  IL21R  CMBL  ROM1  EML3  FHIT  SLC16A7  LOC101928523  VPS28  MYO1F  ADIPOR1  CDH18  CSK  MIR564  TMEM42  DGKZ  CYLD  RFFL  HERC3  KCNIP1  GPR77  LOC101927070  CD34  PRKCQ  MAN1C1  SNORA38  NFKBID  SFRS3  HNRNPH3  RBM45  GSTT1  PPP1R9A  NR2C2  LPP  TNFRSF4  CCL1  RAB27B  CCNB1  DFNB31  LIMCH1  MPP5  NUMB  KLC4  MRPL2  NUP210L  RGMA  MAGI2  SELO  MOV10  GPS1  BSG  NXPE3  CHRM1  ACSL1  DIO1  CACNA2D4  CD164  APBB1IP  TEX2  ATP6V0E1  FOXJ3  ETV4  CLEC17A  ADAM28  FRMD6  POLR2E  CCND2-AS1  PAK6  ADAMTS13  TMEM51  CEACAM21  METTL20  CMC2  HPGDS  RFX1  FAM63A  TMCC2  TGIF1  SUCLA2  CEP350  TMEM45B  PADI4  MBNL2  ERBB2IP  EPB42  DARC  FAM189B  C11orf21  ITSN2  DAZAP2  HK2  CCDC8  MYL9  SPP1  STAT1  TMEM223  JUP  ATXN7L3  DENND4A  AHCYL1  ZNF474  NFIA  HAMP  SCYL3  PDCL  CD53  DLG4  MIB2  CLIP4  LZTS2  TAL1  ANO9  EEF2K  ING4  CXXC5  PIP5K1B  PTPRE  ADARB1  CPT1C  DOK4  SPDYC  ABLIM1  CAMKK1  STOML1  SUV420H1  PDK2  ZNF436  MARCH8  MLC1  IDUA  PTGS1  LLGL2  LOC101929524  SNRNP35  SLC8B1  GPR146  FAR1  WASF2  CABLES2  S1PR4  TEC  SNAPC5  PLAGL1  RAB19  NYNRIN  SORBS1  INPP4A  ACTR3C  LAMA3  DENND2D  IFNL4  DPP8  HNRNPA1L2  SEPP1  HLCS  CLU  DCLK3  ADCY3  GUCY1A3  OLFML3  C22orf34  RXFP4  TM7SF4  SNED1  EMR1  KIF3B  TET2  HDAC9  FARP2  B3GNT5  SGK3  IL23A  GABBR1  GNAZ  CLIP3  KMT2E  DARS  VWA5A  KIAA0753  RNF217-AS1  CTIF  EIF4G3  AMMECR1L  FRMD8  SNORD83A  IRF2  SLFN12  CLK1  TTLL3  TNFRSF8  SLC25A42  AKT2  STK38L  LNPEP  TDRKH  CREG1  VWF  WFIKKN2  MUC1  ZFHX2  ZNF625  MGAT1  SLA  LY6G5C  FAM131A  C6orf134  CD320  ATXN7L3B  C14orf43  VMP1  SNHG11  MIR626  SMARCA4  CPT1A  CD300LB  MICAL3  GPR135  LUZP1  FNDC3B  ECE1  TCF12  LOC100507156  SLC2A9  ATG5  SLC22A18  RUSC1  DHRS11  TMEM200A  FKBP5  DHRS4L2  PAPLN  LOC100128288  PITPNM1  NRIP1  FAM129B  NPLOC4  C6orf47  SLC16A5  GDAP1  HECW1  C20orf27  TTC33  GNG4  CDC42  MGMT  SEMA4A  IKZF1  TBC1D24  TRIM65  MBOAT2  AIF1  SNUPN  CRLF3  LRRC17  CYFIP2  TRIQK  MATR3  AP4S1  LINC01094  AFTPH  DNAJC13  OAS3  MYO9B  RPP14  SLC35B1  CALCOCO1  SNORD124  IL6ST  MICAL2  RAB1B  PHF1  MKL1  ISPD  ADI1  ADCK3  AMT  IL7  SELPLG  ZAP70  BRPF1  HDAC11  SLC24A1  TPM3  KAT6B  NFIX  LTBR  VAMP2  SSFA2  UBA3  RABGEF1  SLC17A2  ZNF319  CEP55  PJA2  HLA-DRA  UBC  IL1RL1  TRHR  TJP2  ATP5E  LINC00299  MRPS26  FBXO9  ZNF865  HIPK1  UAP1  MGA  C20orf195  CD5  NUDT16L1  PTK2B  CHD9  SPSB1  B3GALT5  CDH13  ZNF366  MPV17  DUSP10  SLC25A45  AVIL  IDH1  LTC4S  MOB1B  ABHD13  FAM89B  CHST14  SLC45A3  DRAM1  PCDH9  HDAC4  NAP1L4  C1QTNF6  EIF5A2  HDAC2  FAM158A  ELFN1  GPBP1L1  GGT1  KANK1  PCGF6  MGAT5  EP400  GAMT  LATS2  PLD4  TACSTD2  PITPNA  EGOT  HCG11  IL10RB  EPB41  CCDC97  GPS2  DNA2  ADD3  CCDC149  MIR1234  TBC1D13 |
