# Supplementary material for: DNA methylation dynamic of bone marrow hematopoietic stem cells after allogeneic transplantation
Source: Stem Cell Res Ther. 2019 May 20;10:138. doi: 10.1186/s13287-019-1245-6 (PMC6528331; doi:10.1186/s13287-019-1245-6)
Supplement: Supplementary file 3 — Table S2. Hypo- and Hyper-methylated “stable genes”. (DOC 36 kb) [file 13287_2019_1245_MOESM3_ESM.doc]

**Additional file 3:** **Table S2.** Hypo- and Hyper-methylated “stable genes”.

| Hypo-methylated stable genes | Hyper-methylated stable genes |
| --- | --- |
| MAP3K7CL  TRIM15  BIK  BANK1  SPRED2  SYTL1  PTCRA  ASAP1  MIR194-2  MIR192  PAG1  ANGPT4  MYBPH  SLFN12L  ADARB1  PKIG  MIR650  CNR2  TCONS_00029157  GPR65  FAM113B  OSBPL5  FNDC3B  ZNF710  TRPM2  CHRNA6  SEMA6A  TRIM40  PIK3R5  CEP85L  FSD2  CCDC88B  LITAF  PACSIN1  STRA6  ANKMY1  KLRC1  FXYD2  CCL26  SPON2  ALDH3B2  CPNE3  PXK  C5orf66  ITGB2  ITGA4  C20orf197  GPRIN3  CHST15  ZFHX2  FAM53B  ST5  SIPA1  ZNF385D  PLAT  BOC  ABHD12B  NOX3  FBXO5  CCL3  ADD3  JCHAIN  PARVB  LASP1  SH3BP2  SCML4  IFRD1  GNG4  DFNB31  MIR429  SLC2A9 | PEAK1  PRDM8  CASP8  NDFIP1  ZMIZ1  TP63  PFN3  VARS2  ZNF513  CTBP2  TNK2  NKAPL  NCOR2  TMIGD2  PROM1  FLYWCH1  RNU6-35P  RNU6-64P  SMIM6  C10orf18  INF2  MGMT  TCAP  COL11A2  MIR589  TESPA1  AKNA  SGIP1  KCTD11  ANKRD39  BTNL10  ITPR1  ADCY7  KIAA1539  NR1D2  RC3H2  INPP4A  HOXA5  SNED1  TIAM1  LHX6  ABI2  CRAT  GSTT1  CCDC50  UTS2B  PIK3IP1  CLDN15  ITM2B  KLHDC1  GPR21  MRVI1  TEK  RIN1  SNORD15B  CPT1B  PLEKHA6  RUFY1  RNF185  SLC25A2  CTNNBL1  KIAA0754  CBFA2T2  ZMYND8  HIPK1  GAPVD1  DHRS2  RCBTB1  WDR52  ABCG2  SEC14L1  TNS1  CTSA  DLEU7  PARP11  SEPP1  EIF4G3  FAM65B  PTGS1  MIR126  NAV1  LOC101928304  PRR5L  TPM2  MYO9B  VDR  ZNF668  TNFRSF8  ELMO1  AK1  ZC3H7A  FAM19A2  ZBTB38  TBC1D9B  ZNF577  PCGF3  CILP  TNS3  DLGAP1  NUP210L  MTA2  SLC40A1  HDAC9  STAB1  KRTCAP3  GUCY1B3  ICAM2  SCG5  KLHL5  BACH2  HMGCS1  PAK2  BCL9  CERK  NAPRT1  ACP1  EGF  HOXB3  TNFAIP8  ZBTB4  IL1R1  ZKSCAN4  SNX22  GAPT  CHD6  ABR  RAMP1  RPL10A  ST3GAL1  ADGRG7  LSM12  PPP2R2B-IT1  ANKRD6  CSGALNACT1  PRKAR1B  HOXA4  CXCR6  C2orf88  MAS1L  TNR  BLVRA  TNN  CABLES1  BANP  TMEM204  COMT  OLFML2A  CRHBP  PKM  LDLRAD4  LKAAEAR1  OPRL1  CANT1  FRYL  HIVEP2  AP1B1  MPHOSPH9  TPM4  MS4A5  CDH13  BCAT1  PRR5  C14orf159  SLC20A2  FAM118A  AMIGO3  ABCB6  C1orf70  ZBTB20-AS4  ZBTB20  PRKCQ  INPP4B  HKR1  ERMAP  EPB41  MIR5006  TMCC2  KIAA0748  RGMA  ECI1  RAB32  CCER2  ANKMY1  PDHB  ABLIM1  HIST1H3E  STARD3  DGKD  TBX6  RNF2  ZFP3  SERPINB6  BCMO1  YPEL3  MARCH2  FDX1L  PPFIBP1  RHD |
