# Supplementary material for: DNA methylation dynamic of bone marrow hematopoietic stem cells after allogeneic transplantation
Source: Stem Cell Res Ther. 2019 May 20;10:138. doi: 10.1186/s13287-019-1245-6 (PMC6528331; doi:10.1186/s13287-019-1245-6)
Supplement: Supplementary file 4 — Table S3. Hypo- and Hyper-methylated genes deriving from p2 t4 vs all other t4 patients comparisons. (DOC 33 kb) [file 13287_2019_1245_MOESM4_ESM.doc]

**Supplementary file 4: Table S3.** Hypo- and Hyper-methylated genes deriving from p2 t4 vs all other t4 patients comparisons.

| Hypo-methylated | Hyper-methylated |
| --- | --- |
| ZHX2  MYPN  HSD52  ZNF623  MRPL28  YTHDC2  AGBL3  SBNO2  ESRRB  MTHFR  MRPL28  CYP46A1  ZSCAN4  RBFOX3  APOBEC3A  C16orf68  MIR516B2  PSORS1C1  ERVV-2  MYOC  STAMBPL1  FRMD6  SELPLG  MRPL28  CENPJ  KRTAP25-1  ZNF536  MGC72080  PABPC1  SAP18  TOMM40L  RPA3  TMEM218  ARF5  PITRM1  NOM1  SLC13A4  HEBP2  SLC30A1  ZFP1  FEM1C  NMBR  ZFP69  LIMS1  PBX2  MTDH  ZNF148  TAF2  TMEM74  GRB14  FCRL6  POLI  INTS10  TRIM26  HIVEP2  INTS5  GCNT2  ABR  ABCB6  SLC25A35  BAI3  KDM5B  BRD3  PRKCG  SLC25A37  CHRNA7  ZBTB2  HIST1H4H  ANXA4  CHMP2B  ZNF664  FAF2 | ARID3A  KLHDC8B  NANOS3  TRPM1  C14orf129  NOL10  TBX19  IGFBP5  CATSPER4  IL20RA  PISD  HEPN1  CCDC85B  OR51B2  IRF9  SLMAP  ADPRHL1  EDAR  SH2B3  EHHADH-AS1  COA7  C5orf58  MRPS18A  CCDC71  MYO7B  OR52D1  GPR160  MIEN1  COL15A1  LOC101929622  TMEM61  RASGEF1B  MIR1279  REEP2  KDM4B  LOC101929577  SOLH  TLN2  UBE2U  RNF14  PRND  METTL11B  PVRL4  ZNF517  SLC6A4  POLR2L  CAPN8  ALPPL2  TIAM2  FLYWCH1  LPCAT1  THRSP  SLC38A11  LAMP3  PLXDC1  SNORD115-40  OR2B11  CHST14  PLCG2  NEMP2  RFXAP  C12orf54  LINC01548  LPAR3  FOXN4  RLBP1  KRT32  NODAL  PHLDB1  RBM33  NXN  TCF25  PMM2  PDP2  DAB1  L3MBTL4  FOXC1  HIST1H3A  URI1  C1QTNF8  DHRS4L1  IL1F6  NANP  PIGZ  TSNARE1  BPIL3  RSPH1  OTX1  AHNAK2  PAQR5  LINC01491  SNORD116-30  MED17  KCNK4  FOXK1  NMT2  GSX2  KLF15  ANKH  MEX3B  OAT  RPL26  UCP1  DHRS4L2  NPY5R  ADPRHL2  NRTN  KLF15  GOLGA8B  UBE2Q1  GLI3  TEX2  ANP32B  ANKS6  VWA5B2  CYP2W1  WNT5B  INSL3  LINC01480  SP6  DHRS4L2  KLF15  CALN1 |
